# Characteristics of oral mucositis in patients undergoing haploidentical stem cell transplantation with posttransplant cyclophosphamide: marked difference between busulfan and melphalan regimens

**DOI:** 10.1007/s00520-025-09313-z

**Published:** 2025-03-05

**Authors:** Saki Ogura, Yoshihiko Soga, Hideaki Fujiwara, Rumi Miura, Ken-ichi Matsuoka, Yoshinobu Maeda, Takuo Kuboki

**Affiliations:** 1 Division of Dental Hygienist, Okayama University Hospital, Okayama, Japan; 2 Division of Hospital Dentistry, Okayama University Hospital, Okayama, Japan; 3 Department of Hematology and Oncology, Okayama University Hospital, Okayama, Japan; 4 Department of Hematology, Oncology and Respiratory Medicine, Graduate School of Medicine, Dentistry and Pharmaceutical Sciences, Okayama University, Okayama, Japan; 5 Department of Oral Rehabilitation and Regenerative Medicine, Graduate School of Medicine, Dentistry and Pharmaceutical Sciences, Okayama University, Okayama, Japan

**Keywords:** Oral mucositis, Hematopoietic cell transplantation, Posttransplant cyclophosphamide, Busulfan, Melphalan

## Abstract

**Purpose:**

This study was performed to examine the effects of conditioning regimens on oral mucositis in haploidentical (haplo) donor hematopoietic stem cell transplantation (HSCT) with posttransplant cyclophosphamide (PTCy).

**Methods:**

Thirty consecutive patients (male, 23; female, 7; 18–68 years, median, 59 years) undergoing haplo-HSCT with PTCy using one of three conditioning regimens—reduced intensity conditioning (RIC)-melphalan (Mel); RIC-Busulfan (Bu); and myeloablative conditioning (MAC)-Bu—were enrolled in this study. Data on the WHO grade of oral mucositis (day − 7 to + 20) were collected retrospectively. The incidences of ulcerative and severe mucositis (Grade 2–4 and Grade 3–4, respectively) were compared between the three groups.

**Results:**

Ulcerative mucositis occurred in 0% (0/10) of patients in the RIC-Mel group, 57.1% (4/7) in the RIC-Bu group, and 100% (13/13) in the MAC-Bu group. The differences between the RIC-Mel and RIC-Bu groups and between the RIC-Bu and MAC-Bu groups were significant (all *P* < 0.05). Severe mucositis occurred in 57.1% (4/7) of patients in the RIC-Bu group and 100% (13/13) of patients in the MAC-Bu group, and the difference was significant (*P* < 0.05). The rates of ulcerative mucositis (≥ grade 2) and of severe mucositis (≥ grade 3) were significantly higher in the MAC-Bu group than the RIC-Bu group on days 10, 13, 15, and 16 and on days 10, 14, 15, and 16, respectively (all *P* < 0.05).

**Conclusion:**

The risk of oral mucositis in patients undergoing haplo-HSCT with PTCy is highest with the MAC-Bu conditioning regimen, followed by RIC-Bu, and lowest with RIC-Mel.

**Supplementary Information:**

The online version contains supplementary material available at 10.1007/s00520-025-09313-z.

## Introduction

Oral mucositis is a common complication in hematopoietic stem cell transplantation (HSCT) [1]. Severe oral mucositis significantly reduces quality of life and increases the treatment-related mortality risk in patients undergoing HSCT [1, 2]. Oral mucositis is associated with various issues, including pain, difficulty with eating and speaking, inability to take medications, prolonged hospital stay, the need for parenteral nutrition, and high risks of infection and sepsis [3, 4].

To better manage oral mucositis, it is essential to understand its severity and clinical course during HSCT. Theoretically, the intensity of the conditioning regimen should be correlated with the degree of oral mucositis. However, a systematic review by Chaudhry et al. showed that reduced-intensity conditioning (RIC) regimens led to a high frequency of oral mucositis comparable to that seen in myeloablative regimens [5], although some reports suggested that RIC regimens are associated with a lower rate of severe oral mucositis [6, 7]. These discrepancies were likely due to the lack of standardization of various confounding factors, including the many types of regimens used for conditioning and graft-versus-host disease (GVHD) prophylaxis, HLA matching status, donor stem cell source, and local oral condition with/without dental treatment before HSCT and oral management during HSCT. Therefore, it is challenging to identify risk factors by analyzing associations between each conditioning regimen and the severity of oral mucositis.

Significant progress has been made in allogeneic HSCT, allowing the clinical use of haploidentical HSCT (haplo-HSCT) with posttransplant cyclophosphamide (PTCy) and calcineurin inhibitors [8–10]. Previously, we reported the clinical course and degree of oral mucositis in patients receiving RIC regimens for allogeneic HSCT [11]. We also reported progress in oral care and reduction of oral mucositis by multidisciplinary intensive oral management [12]. During our ongoing activities, we have observed many patients with less severe mucositis in haplo-HSCT with PTCy. A recent systematic review and meta-analysis conducted by Al-Jamaei revealed that the risk of severe oral mucositis is higher in patients receiving methotrexate (MTX) compared to other approaches to prevent GVHD [13]. Therefore, haplo-HSCT with PTCy, which does not include posttransplant MTX administration, would exhibit a characteristic trend in the clinical course of oral mucositis. However, few studies specifically evaluated the severity of oral mucositis as a primary end point based on the conditioning regimen used in haplo-HSCT with PTCy.

This study was performed to examine the effects of conditioning regimens on oral mucositis in haplo-HSCT with PTCy.

## Subjects and methods

This single-center retrospective study was performed at the Division of Hospital Dentistry and the Department of Hematology and Oncology, Okayama University Hospital. The institutional ethics committee approved this study (Ken 2309–018). The procedures in human participants were performed in accordance with the Declaration of Helsinki. Informed consent was obtained by following the Ethical Guidelines for Medical and Biological Research Involving Human Subjects of the Japanese Ministries.

### Subjects

Thirty consecutive patients undergoing haplo-HSCT with PTCy with the conditioning regimens described below and discharged from a protective environment after engraftment between September 2021 and December 2023 were enrolled in the study.

#### Conditioning regimens and GVHD prophylaxis

One of three conditioning regimens was applied at the discretion of the attending physician taking the disease status and condition of the patient into consideration:

1. RIC-melphalan (Mel) consisted of fludarabine (Flu) (180 mg/m^2^; 30 mg/m^2^ from day − 7 to − 2), Mel (80 mg/m^2^; 40 mg/m^2^ on days − 3 and − 2), and total body irradiation (TBI) (2 Gy; 2 Gy × 1 on day − 1) [14–16]. Dose changes of Mel (50–100 mg/m^2^; 25–50 mg/m^2^ on days − 3 and − 2) and TBI (2 or 4 Gy; 2 Gy × 1 or 2 on day − 1) were allowed at the physician’s discretion.
2. RIC-Bu consisted of Flu (150 mg/m^2^; 30 mg/m^2^ from day − 6 to − 2), Busulfan (Bu) (6.4 mg/kg; 3.2 mg/kg on days − 6 and − 5), and TBI (4 Gy; 2 Gy × 2 on day − 1) [17, 18].
3. Myeloablative conditioning (MAC)-busulfan (Bu) consisted of Flu (150 mg/m^2^; 30 mg/m^2^ from day − 6 to − 2), Bu (12.8 mg/kg; 3.2 mg/kg from day − 6 to − 3), and TBI (4 Gy; 2 Gy × 2 on day − 1) [17].

The standard PTCy regimen consisted of PTCy at 50 mg/kg/day on days + 3 and + 4, and tacrolimus (Tac) initiating on day + 5. Dose changes of PTCy (40 or 50 mg/kg/day) and changes to the day of initiation of Tac (from day − 1 or day + 5) were allowed at the discretion of the attending physician. All patients received mycophenolate mofetil (MMF) at a dose of 15 mg/kg orally twice daily from day + 5 to + 30, with a maximum daily dose of 2000 mg.

#### General infection control

Fluoroquinolone was administered orally for prophylaxis against bacterial infection, and micafungin and posaconazole were administered until the end of PTCy administration for prophylaxis against fungal infection. Prophylaxis was also provided against herpes virus infection with acyclovir and against cytomegalovirus reactivation with letermovir. Neutropenic fever was managed as reported previously [19]. Briefly, empirical antibiotic therapy was administered promptly in all neutropenic patients at the onset of fever and in afebrile patients who were neutropenic but who had signs or symptoms compatible with infection. A fourth-generation cephalosporin (*e.g.*, cefepime) or carbapenem (*e.g.*, meropenem) was administered intravenously as empirical antibiotic therapy. G-CSF (lenograstim 5 g/kg/day or filgrastim 300 g/m^2^) was given intravenously for 60 min starting on day 1 or 5 and was continued until the absolute neutrophil count exceeded 500/L.

### Oral management

Intensive oral care was performed for all patients receiving HSCT as described in our previous reports with minor modifications [11, 12]. Before HSCT, all patients scheduled to undergo HSCT were referred to dentists, and necessary dental treatment was performed if the patient’s general condition allowed. Measures such as grinding the sharp edges of the teeth and restorations that may lead to ulcer formation were also taken. All subjects received instruction regarding self-management of oral hygiene; tooth brushing with soft bristles after every meal and gentle oral rinsing with 0.0048% azulene sodium sulfonate and 12% glycerin solution every 2 h during the day were also indicated. Chlorhexidine could not be used due to a warning from the Ministry of Health, Labour and Welfare, Japan, regarding the risk of allergic anaphylaxis. To protect the oral mucosa, episil® oral liquid (Solasia Pharma K. K., Tokyo, Japan) and petroleum jelly were used as appropriate. Nurses, dental hygienists, and dentists performed oral management in patients with poor condition. When brushing was impossible in patients with severe mucositis, dental hygienists gently wiped out the dental and mucosal plaque using cotton swabs wet with normal saline solution to minimize the presence of any visible dental plaque.

#### Oral cryotherapy

Following the recommendations of the MASCC/ISOO Clinical Practice Guidelines for the Management of Mucositis [20, 21], patients undergoing conditioning regimens with Mel received oral cryotherapy based on the description of Mori et al. [22]. Patients were instructed to place ice chips in their mouth 15 min before, during (30–60 min), and for an additional 30 min after Mel infusion.

#### Assessment of oral mucositis

The degree of oral mucositis in patients undergoing HSCT was assessed daily using the World Health Organization (WHO) grading system [23]. This system is recommended for evaluating oral mucositis in HSCT patients, as described in the guidance on mucositis assessment from the MASCC Mucositis Study Group and ISOO [24]. The criteria for oral mucositis were as follows:

Grade 0: No change.

Grade 1: Soreness/erythema.

Grade 2: Erythema, ulcers; can eat solids.

Grade 3: Ulcers; requires liquid diet only.

Grade 4: Alimentation not possible.

Nurses, dental hygienists, and dentists conducted assessments as part of their daily clinical routines. The dental professionals performed oral assessments and management at least once a week, and more frequently (2–5 days per week) in cases with mucositis. They also ensured their assessments were consistent with those of the nurses. When the assessments of dental professionals and nurses differed, the evaluation by the dental professionals was prioritized. However, the evaluations of both dental professionals and nurses were examined in detail and judged accordingly.

#### Statistical analysis

Comparisons between two groups were performed using Fisher’s exact test. Comparisons of quantitative data among three groups were performed using the Kruskal–Wallis and Dunn-Bonferroni tests. Comparisons of qualitative data among three groups were performed using the Fisher-Freeman-Halton Exact Test. Statistical analyses were performed using IBM® SPSS® Statistics 29 (IBM, Armonk, NY, USA). In all analyses, *P* < 0.05 was taken to indicate statistical significance.

## Results

### Patient characteristics

Patient characteristics are shown in Table 1. The total study population consisted of 23 males and 7 females with a median age of 59 years (18–68 years). There was a significant difference in age distribution between the RIC-Mel, RIC-Bu, and MAC-Bu groups (*P* = 0.001, Kruskal–Wallis test). The RIC-Bu group was significantly older than the RIC-Mel and MAC-Bu groups (both *P* = 0.002, Dunn-Bonferroni test). The median (range) time to engraftment was 15 (12–28) days. There were no significant differences in distribution of PTCy dose (standard or reduced), timing of Tac initiation (day − 1 or + 5), or days to engraftment between these groups.

**Table 1:** Patient characteristics by conditioning regimen and GVHD prophylaxis

	All	RIC-Mel	RIC-Bu	MAC-Bu	*P*
Number of subjects	30	10	7	13	
Median age (range), (years)	59 (18–68)	58.5 (29–62)	65 (64–68)	57 (18–65)	0.001*^a^
Diagnosis at transplantation, no. (%)					
AML	14 (46.7)	3 (30.0)	6 (85.7)	5 (38.5)	
ALL	5 (16.7)	3 (30.0)		2 (15.4)	
MDS	8 (26.7)	1 (10.0)	1 (14.3)	6 (46.2)	
DLBCL	1 (3.3)	1 (10.0)			
ATLL	1 (3.3)	1 (10.0)			
PTCL	1 (3.3)	1 (10.0)			
post-PTCy dose (day + 3 to day + 4, mg/kg/day)					
50–50	17 (56.7)	6 (60.0)	2 (28.6)	9 (69.2)	0.263^b,c^
50–40	1 (3.3)		1 (14.3)		
40–40	12 (40.0)	4 (40.0)	4 (57.1)	4 (30.8)	
Day of Tac initiation (days relative to HSCT)					
− 1	10 (33.3)	4 (40.0)	3 (42.9)	3 (23.1)	0.687^b^
+ 5	20 (66.7)	6 (60.0)	4 (57.1)	10 (76.9)	
Days to engraftment (range), days	15 (12–28)	15 (14–18)	16 (12–19)	16 (14–28)	0.481^a^

ALL, acute lymphoblastic leukemia; AML, acute myeloid leukemia; ATLL, adult T-cell leukemia-lymphoma; Bu, Busulfan; Cy, cyclophosphamide; DLBCL, diffuse large B-cell lymphoma; HSCT, hematopoietic stem cell transplantation; MAC, myeloablative-conditioning; MAC-Bu, Flu (150 mg/m^2^)/Bu (12.8 mg/kg)/TBI (4 Gy) group; MDS, myelodysplastic syndrome; Mel, melphalan; PTCL, peripheral T-cell lymphoma; RIC, reduced-intensity conditioning; RIC-Bu, Flu (150 mg/m^2^)/Bu (6.4 mg/kg)/TBI (2 Gy) group; RIC-Mel, Fludarabine (Flu) (180 mg/m^2^)/Mel (50–100 mg/m^2^)/total body irradiation (TBI) (2 or 4 Gy) group; Tac, tacrolimus

^*^P < 0.05. ^a^ Kruskal–Wallis test. ^b^ Fisher-Freeman-Halton exact test. ^c^ Comparison between the frequency with the standard dose (50–50 mg/kg/day) and other reduced doses

Changes to the dose of Mel and TBI were allowed at the physician’s discretion only in the RIC-Mel group, as described in the Methods section. Mel doses were 50 mg/m^2^ (Mel 50), 80 mg/m^2^ (Mel 80), and 100 mg/m^2^ (Mel 100), and TBI dose was 2 or 4 Gy. The combination and numbers of patients were as follows: Flu/Mel 50/TBI 2 Gy, *n* = 1; Flu/Mel 80/TBI 2 Gy, *n* = 3; Flu/Mel 80/TBI 4 Gy, *n* = 3; Flu/Mel 100/TBI 2 Gy, *n* = 3.

### Oral mucositis in all subjects

**Fig. 1:**
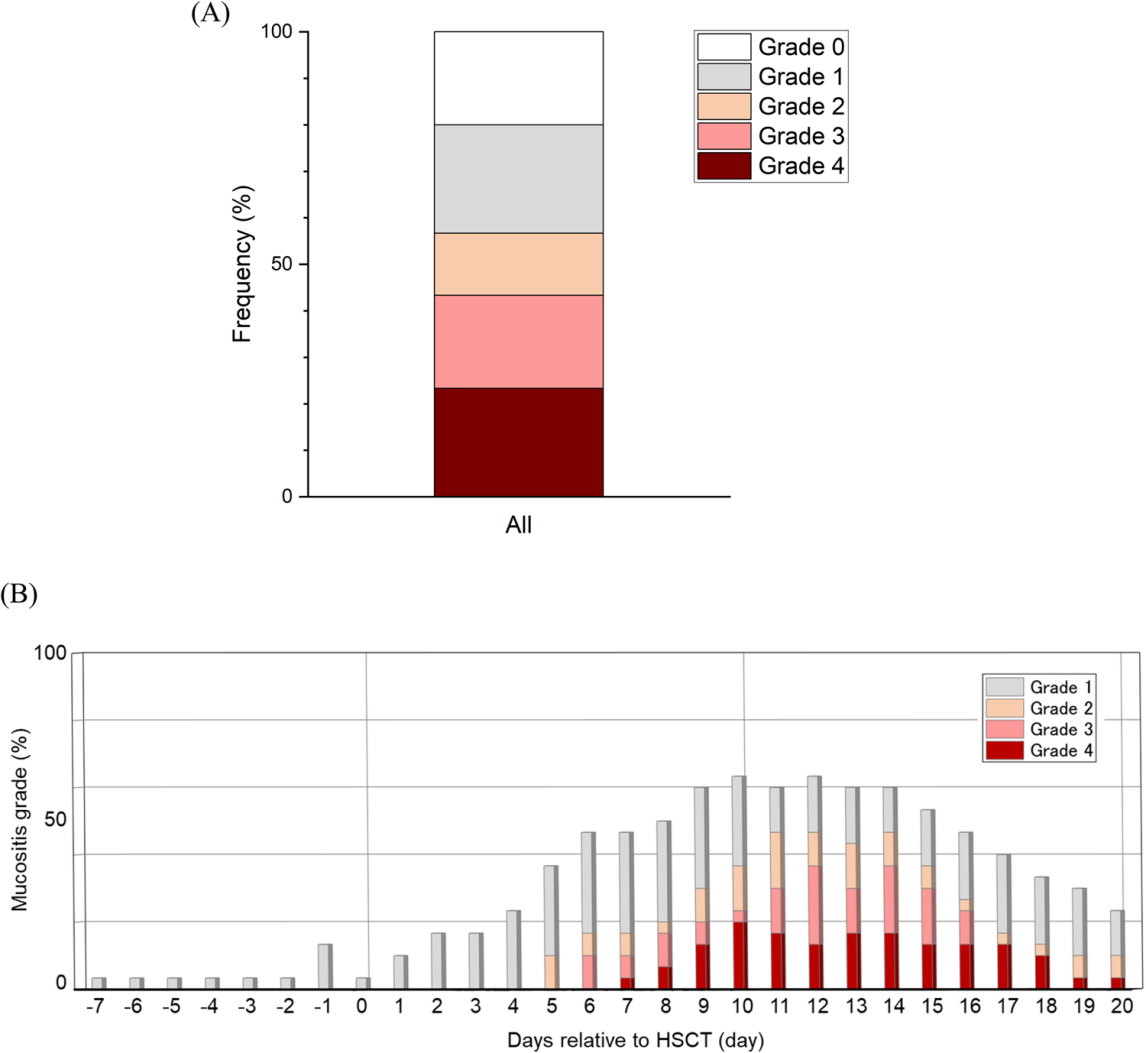
Oral mucositis in all subjects. A: Frequency of the highest grade of oral mucositis during the evaluation period. Ulcerative mucositis (≥ grade 2) occurred in 56.7% (17/30) of patients, and severe mucositis (≥ grade 3) appeared in 43.3% (13/30) of patients. B: The clinical course of oral mucositis in all patients. The severity of oral mucositis increased, peaking between days 11 and 15 with grade 3 or higher, and subsequently decreased. The earliest day of ulcerative oral mucositis was day 5

The frequency of the highest grade and the clinical course of oral mucositis in all subjects during the study period are shown in Fig. 1. Ulcerative mucositis (≥ grade 2) was seen in 56.7% (17/30) of patients, and severe mucositis (≥ grade 3) occurred in 43.3% (13/30) of patients (Fig. 1A). The severity of oral mucositis increased, peaking between 11 and 15 days with grade 3 or higher, and subsequently decreased (Fig. 1B). The earliest occurrence of ulcerative oral mucositis was on day 5 (Fig. 1B).

### Oral mucositis in RIC-Mel and RIC-Bu groups

**Fig. 2:**
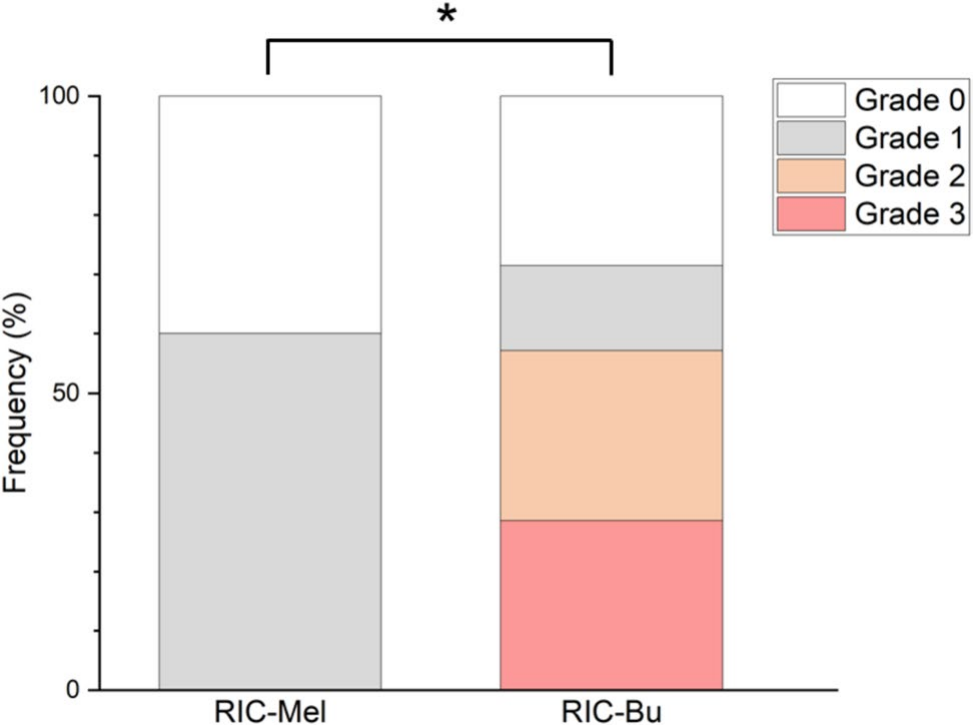
Oral mucositis in RIC-Mel and RIC-Bu groups. Frequency of the highest grade of oral mucositis during the evaluation period. Ulcerative mucositis (≥ grade 2) and severe mucositis (≥ grade 3) were only seen in the RIC-Bu group at rates of 57.1% (4/7) and 28.6% (2/7), respectively. There were no cases of ulcerative mucositis among patients treated with the RIC-Mel regimen (0/10). The incidence of ulcerative mucositis (≥ grade 2) was significantly different between the RIC-Bu group and the RIC-Mel group (*P < 0.05, Fisher’s exact test). The difference in severe mucositis (≥ grade 3) was not significant

The frequencies of the highest grade of oral mucositis in the RIC-Mel and RIC-Bu groups during the study period are shown in Fig. 2. Ulcerative mucositis (≥ grade 2) and severe mucositis (≥ grade 3) were only seen in the RIC-Bu group with rates of 57.1% (4/7) and 28.6% (2/7), respectively (Fig. 2). Despite the same RIC regimen, the Bu regimen was associated with ulcerative/severe mucositis, while there were no cases of ulcerative mucositis among patients treated with the RIC-Mel regimen. A significant difference was observed in ulcerative mucositis (≥ grade 2) between the RIC-BU group and the RIC-Mel group (*P* < 0.05). The difference in severe mucositis (≥ grade 3) was not significant. The daily frequencies of ulcerative/severe mucositis (≥ grade 2/3) tended to be higher in the RIC-Bu group than the RIC-Mel group, in which no ulcerative mucositis occurred during the observation period, but the differences were not significant.

### Oral mucositis in RIC-Bu and MAC-Bu groups

**Fig. 3:**
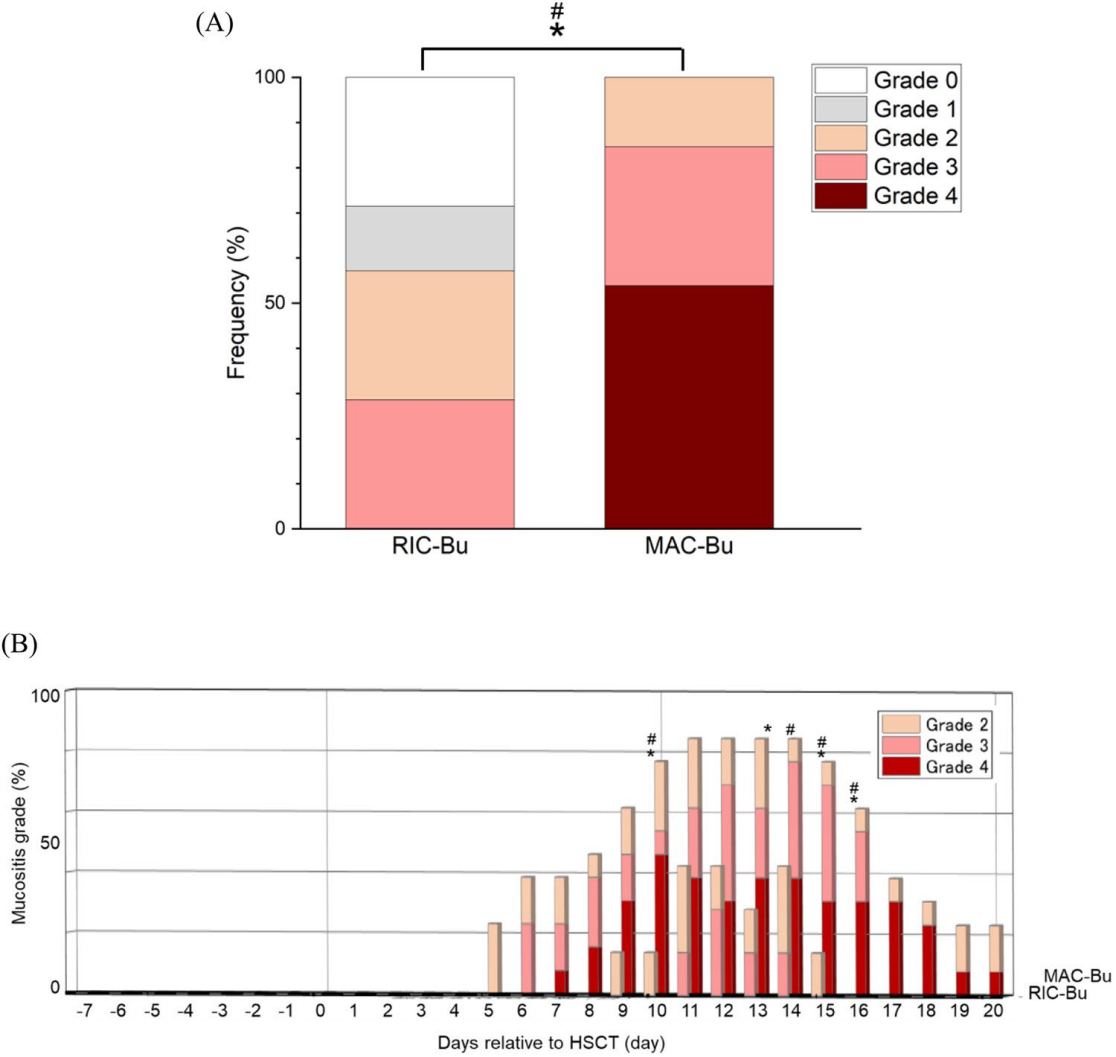
Oral mucositis in RIC-Bu and MAC-Bu groups. A: Frequency of the highest grade of oral mucositis during the evaluation period. The frequencies of ulcerative mucositis (≥ grade 2) were 57.1% (4/7) in the RIC-Bu group and 100% (13/13) in the MAC-Bu group, and the difference was significant (*P < 0.05, Fisher’s exact test). The frequencies of severe mucositis (≥ grade 3) were 28.6% (2/7) in the RIC-Bu group and 84.6% (11/13) in the MAC-Bu group, and the difference was significant (^#^*P* < 0.05, Fisher’s exact test). B: Clinical courses of ulcerative (≥ grade 2) and severe (≥ grade 3) oral mucositis in RIC-Bu (*n* = 7) and MAC-Bu (*n* = 13) groups. Daily frequencies of ulcerative mucositis (≥ grade 2) were significantly higher in the MAC-Bu group than the RIC-Bu group on days 10, 13, 15, and 16 (*P < 0.05, Fisher’s exact test). Daily frequencies of severe mucositis (≥ grade 3) were significantly higher in the MAC-Bu group than the RIC-Bu group on days 10, 14, 15, and 16 (^#^*P* < 0.05, Fisher’s exact test)

The frequencies of the highest grade and clinical course of oral mucositis in RIC-Bu and MAC-Bu groups during the study period are shown in Fig. 3. The frequencies of the highest grade during the study period were as follows. Ulcerative mucositis (≥ grade 2) occurred in 57.1% (4/7) of patients in the RIC-Bu group and 100% (13/13) of patients in the MAC-Bu group (*P* < 0.05) (Fig. 3A). Severe mucositis (≥ grade 3) occurred in 28.6% (2/7) of patients in the RIC-Bu group and 84.6% (11/13) of patients in the MAC-Bu group (*P* < 0.05). Bu showed a dose-dependent influence on severity of oral mucositis. Daily frequencies of ulcerative mucositis (≥ grade 2) were significantly higher in the MAC-Bu group compared with the RIC-Bu group on days 10, 13, 15, and 16 (all *P* < 0.05) (Fig. 3B). Daily frequencies of severe mucositis (≥ grade 3) were significantly higher in the MAC-Bu group than the RIC-Bu group on days 10, 14, 15, and 16 (all *P* < 0.05).

## Discussion

Although Mel has been reported to cause severe oral mucositis, the most notable result of this study was the absence of ulcerative oral mucositis presenting as ulcers in the RIC-Mel group. In addition, we showed that Bu had a dose-dependent influence on the severity of oral mucositis.

Mel is a cytotoxic alkylating agent [25] for which mucositis is a well-known nonhematological dose-limiting toxicity [26]. However, no ulcerative oral mucositis was observed in the present study. In haplo-HSCT with PTCy, oral mucositis was not worsened by post-HSCT MTX administration, which may have contributed to this result. In a recent study performed in Australia, Nakagaki et al. reported low rates of severe oral mucositis (WHO grade 3–4) and ulcerative oral mucositis (WHO grade 2–4) of 19% and 41%, respectively, in PTCy (100 mg/kg) HSCT with Flu (160 mg/m^2^), Mel (100 mg/m^2^), and TBI (2 Gy) [27]. This reduced frequency of ulcerative oral mucositis in haplo-HSCT with PTCy using RIC-Mel was consistent with our observations, while our results showing no ulcerative mucositis were clear. This was likely because the dose of Mel used at our institute is lower, with a base dose of 80 mg/m^2^ (range 50–100 mg/m^2^).

The frequencies of ulcerative mucositis were 100% in the MAC-Bu group and 57.1% in the RIC-Bu group, and this difference was significant. The only difference in treatment regimen between these two groups was the dose of Bu (12.8 mg/kg and 6.4 mg/kg, respectively). Furthermore, oral mucositis occurred in the RIC-Bu group, but was absent in the RIC-Mel group. Taken together, these results suggest that Bu plays a role in determining the severity of oral mucositis.

As no ulcerative mucositis was observed in haplo-HSCT with PTCy using RIC-Mel (Flu/Mel/TBI) conditioning, the doses for Flu and TBI used at our institute (180 mg/m^2^ and 2–4 Gy, respectively) were also confirmed to have a low risk of inducing oral mucositis. Indeed, a previous study showed that conditioning with TBI alone at a dose of 2 Gy did not cause regimen-related painful mucositis [28]. Flu acts synergistically with radiation to kill tumor cells [29]. However, the impact of TBI at a low dose of 2–4 Gy will likely be limited for oral mucositis.

This study had some limitations. Mel doses were low and inconsistent. All of our Mel regimens were based on the regimen reported by Brammer et al. [15], and dose modifications of Mel (50–100 mg/m^2^) as a RIC regimen were frequently utilized at the discretion of the attending physician based on the condition of the patient at our institution. This RIC (low-dose) Mel regimen in haplo-HSCT with PTCy is becoming increasingly common, and our results will serve as reference data. Another limitation was the retrospective nature of this study. However, to use the data for clinical research in the future, dentists and dental hygienists have been participating in the routine clinical evaluation of oral mucositis with high accuracy in our ward since our previous reports [11, 12]. In addition, the number of subjects included in this study was small. We perform HSCT in about 50–60 patients per year. However, there are many different regimens for HSCT, and focusing on specific regimens to eliminate confounding factors limits the sample size. Factors potentially associated with oral mucositis, such as disease status, oral health prior to conditioning, and systemic diseases, were not taken into account due to the small sample size. Therefore, we are planning to conduct a prospective multicenter study in the near future based on the present study.

In conclusion, the risk of oral mucositis in patients undergoing haplo-HSCT with PTCy was highest with the MAC-Bu conditioning regimen, followed by RIC-Bu, and lowest with RIC-Mel.

## Supplementary Information

Below is the link to the electronic supplementary material.

ESM 1 (XLSX 15.0 KB)

## Data Availability

Data is provided within the manuscript or supplementary information files.
